# DXA-Derived Indices in the Characterisation of Sarcopenia

**DOI:** 10.3390/nu14010186

**Published:** 2021-12-31

**Authors:** Natascha Schweighofer, Caterina Colantonio, Christoph W. Haudum, Barbara Hutz, Ewald Kolesnik, Ines Mursic, Stefan Pilz, Albrecht Schmidt, Vanessa Stadlbauer, Andreas Zirlik, Thomas R. Pieber, Nicolas Verheyen, Barbara Obermayer-Pietsch

**Affiliations:** 1 Department of Internal Medicine, Division of Endocrinology and Diabetology, Medical University of Graz, 8036 Graz, Austria; christoph.haudum@medunigraz.at (C.W.H.); barbara.hutz@medunigraz.at (B.H.); ines.mursic@medunigraz.at (I.M.); stefan.pilz@medunigraz.at (S.P.); thomas.pieber@medunigraz.at (T.R.P.); barbara.obermayer@medunigraz.at (B.O.-P.); 2 CBmed, Center for Biomarker Research in Medicine, 8010 Graz, Austria; vanessa.stadlbauer@medunigraz.at; 3 Department of Internal Medicine, Division of Cardiology, Medical University of Graz, 8036 Graz, Austria; caterina.colantonio@medunigraz.at (C.C.); ewald.kolesnik@medunigraz.at (E.K.); albrecht.schmidt@medunigraz.at (A.S.); andreas.zirlik@medunigraz.at (A.Z.); nicolas.verheyen@medunigraz.at (N.V.); 4 Department of Internal Medicine, Division of Gastroenterology and Hepatology, Medical University of Graz, 8036 Graz, Austria

**Keywords:** DXA, DXA-derived muscle mass indices, BioPersMed cohort

## Abstract

Sarcopenia is linked with increased risk of falls, osteoporosis and mortality. No consensus exists about a gold standard “dual-energy X-ray absorptiometry (DXA) index for muscle mass determination” in sarcopenia diagnosis. Thus, many indices exist, but data on sarcopenia diagnosis agreement are scarce. Regarding sarcopenia diagnosis reliability, the impact of influencing factors on sarcopenia prevalence, diagnosis agreement and reliability are almost completely missing. For nine DXA-derived muscle mass indices, we aimed to evaluate sarcopenia prevalence, diagnosis agreement and diagnosis reliability, and investigate the effects of underlying parameters, presence or type of adjustment and cut-off values on all three outcomes. The indices were analysed in the BioPersMed cohort (58 ± 9 years), including 1022 asymptomatic subjects at moderate cardiovascular risk. DXA data from 792 baselines and 684 follow-up measurements (for diagnosis agreement and reliability determination) were available. Depending on the index and cut-off values, sarcopenia prevalence varied from 0.6 to 36.3%. Height-adjusted parameters, independent of underlying parameters, showed a relatively high level of diagnosis agreement, whereas unadjusted and adjusted indices showed low diagnosis agreement. The adjustment type defines which individuals are recognised as sarcopenic in terms of BMI and sex. The investigated indices showed comparable diagnosis reliability in follow-up examinations

## 1. Introduction

The increasing number of persons older than 60 years make sarcopenia an increasing problem for healthcare systems and societies since it increases physical frailty, disability [1] and hospitalisation risk [2], leading to long-term care placement [3] and increased mortality [4,5,6,7]. The prevalence of sarcopenia is amplified in the case of malnutrition, nutritional deficiencies and chronic diseases, such as cardiovascular disease, diabetes or liver cirrhosis, all of which are often seen in the elderly [8,9,10].

The definition of sarcopenia is based on two pillars: low muscle mass and low muscle function (strength or performance or both) [11]. During ageing, a loss of muscle mass of 8% per decade, starting at the age of 40 years, increases to 15% after 70 years of age. This occurs together with a decline in muscle strength, declining at a faster rate due to changed muscle quality [12,13]. Muscle mass can be determined by computed tomography, magnetic resonance imaging or spectroscopy, ultrasound and bioelectrical impedance analysis and dual-energy X-ray absorptiometry (DXA), the gold standard for body composition analysis [14,15]. DXA is also advised by the revised EWGSOP guidelines [4] for sarcopenia confirmation in practice.

Several DXA-derived muscle mass indices differing in the prediction of sarcopenia prevalence have been established. Only the impact of cut-off values on differences in sarcopenia prevalence is well known. Information regarding the impact of adjusting DXA-derived muscle mass indices, sarcopenia diagnosis agreement (often assuming that the same persons are diagnosed as sarcopenic), diagnosis reliability and possible influencing factors are lacking.

Since pharmacological approaches were not convincing, sarcopenia therapy is limited to a combination of exercise and protein supplementation [16,17]. To improve dietary muscle support, various macro- and micronutrients are under investigation. To determine the effects of new interventions and to allow an early start of intervention/therapy measures, a reliable determination of muscle mass changes and sarcopenia diagnosis in all sexes and weight groups is essential.

The aim of the present study was to determine the prevalence of sarcopenia in an asymptomatic middle-aged cohort with at least one cardiovascular risk factor using nine DXA-derived indices. To further examine the differences in the prevalence of sarcopenia by determining the agreement of sarcopenia diagnosis between the indices and to investigate influencing factors such as underlying parameters of indices, absence/presence or type of adjustment, vitamins D and K and calcium metabolism are influencing factors. Furthermore, we aimed to evaluate the reliability of sarcopenia diagnosis with follow-up data.

## 2. Materials and Methods

### 2.1. Participants, Study Description and Definition of Comorbidities

Data were generated in a population-based, single-centre, prospective, observational study, the ”Biomarkers of Personalized Medicine“ cohort (BioPersMed), to evaluate novel biomarkers for the assessment of cardiovascular and metabolic disease and related complications. The study cohort consisted of asymptomatic subjects without manifested cardiovascular disease (CVD), but at least one classical risk factor for CVD defined by the European Society of Cardiology. A total of 1022 community-dwelling adult persons were recruited by the Divisions of Cardiology and Endocrinology and Diabetology at the Medical University of Graz. Written informed consent was obtained from each participant and the study was approved by the institutional review board of the Medical University of Graz (EK-number 24-224 ex 11/12). The BioPersMed study was conducted in compliance with Good Clinical Practice Guidelines Procedures (GCP) and complied with the Declaration of Helsinki and Austrian laws. The design of the BioPersMed study, data management, biobanking and data analyses are compliant with the STROBE, STROBE-ME and STREGA recommendations.

### 2.2. Anthropometric Measurements

Bodyweight was determined using an electronic scale and height was measured using a fixed stadiometer; BMI was calculated as weight in kilograms divided by height in meters squared (kg/m^2^). BMI classification was conducted according to WHO guidelines. In our study, all persons with a BMI < 25 kg/m^2^ were considered normal weight.

### 2.3. DXA Measurements

Bone density as well as regional body composition were measured via dual-energy X-ray absorptiometry (DXA) using the Lunar iDXA system (GE Healthcare GmbH, Vienna, Austria). DXA data from the baseline (*n = 792*) and the consecutive clinical consultation after two years *(n = 684*) were used for analysis. Least significant change of the BMD (femur and lumbar spine) was calculated at 95% precision: root squared mean of 0.030/0.026 g/cm^2^, respectively, with a CV of 0.024/0.032 g/cm^2^, respectively (2.4/3.2 percent).

In this study, the definition of sarcopenia is based on muscle mass without the inclusion of information regarding muscle function in both the baseline and 2 years follow up data. Seven muscle mass indices were selected; rLM and ALMI were analysed with two different cut-off points creating rLM20 and rLMABC, as well as ALMI NM and ALMI Min, totalling the number of investigated indices to nine (see Table 1). ASM (appendicular skeletal muscle mass) and AMMI (appendicular skeletal muscle mass index) were selected, as they are the EWGSOP2 recommended DXA parameters for sarcopenia diagnosis. ALMI (appendicular lean mass index) is used by the Foundation for the National Institutes of Health (FNIH) Sarcopenia Project to determine low muscle mass. rLM (relative lean mass) not only combines adjustment to fat mass and height but also compromises an alternative approach to gain cut-off values for a young reference cohort. TSMI (total skeletal muscle mass index) was selected due to the use of total skeletal muscle mass as a basis. SMI (skeletal muscle mass index) was chosen for its adjustment to weight and LESMI (lower extremity skeletal muscle mass index) since a similar parameter can be calculated based on CT measurements, thus enabling a comparison of these two techniques [18]. All cut-off values are summarised in Table 1.

**Figure 1:**
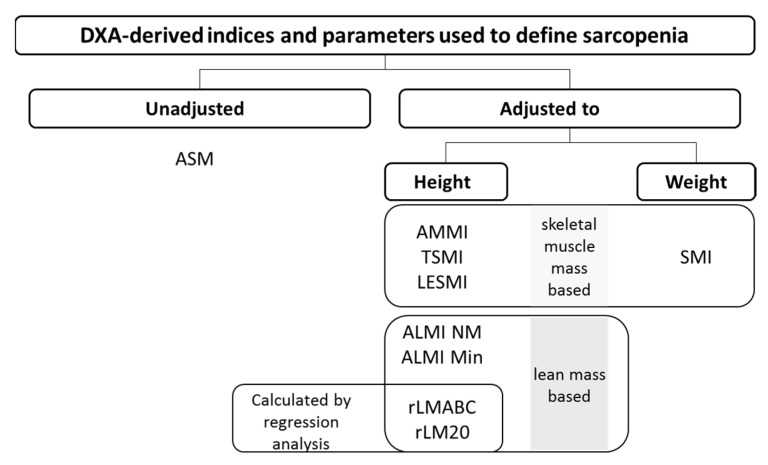
Allocations of indices to adjustment or baseline parameter for calculation. DXA: dual-energy X-ray absorptiometry; ASM: appendicular skeletal muscle mass; AMMI: appendicular skeletal muscle mass index; TSMI: total skeletal mass index; LESMI: lower extremity skeletal muscle mass index; SMI: skeletal muscle mass index; ALMI: appendicular lean mass index; Min: reference values Minnesota; NM: reference values New Mexico; rLMABC: relative lean mass (20th percentile ABC Health study); rLM20: relative lean mass (20th percentile).

DXA-derived indices investigated in this study can be allocated to either the parameters they are based on (parameter of origin) (skeletal muscle mass or lean mass) or the adjustment used for comparability (height or weight). For details, see Figure 1.

### 2.4. Statistical Analysis

Data are expressed as mean ± SD unless noted otherwise. Differences between groups were analysed via either a 2-tailed unpaired Student’s t-test or the Kruskal–Wallis test according to the distribution of data (SPSS Software version 25 (IBM Corp., New York, NY, USA). A *p*-value < 0.05 was considered significant.

Cohen’s Kappa (κ) and Fleiss‘ Kappa [26,27] were used to determine the level of agreement between indices and to evaluate the reliability of sarcopenia definition in the consecutive clinical presentation. Binary logistic regression models were used to determine factors influencing the diagnosis of sarcopenia by different DXA-derived indices, including age, sex, alcohol consumption (drinks per week), smoking (pack years), fat mass (kg), BMI, height (m), number of comorbidities, vitamin D deficiency, parathyroid hormone (PTH), vitamin K levels, genetically determined hypolactasia and lifestyle. The lifestyle parameter was created by factor analysis and consists of the ergospirometry parameters VO_2_max, anaerobic threshold (Watt), maximum heart frequency (1/min), respiratory quotient (max) and maximum loading (Watt). Vitamin D deficiency was defined as season-adjusted 25-OH vitamin D levels lower than 20 ng/mL. 25-hydroxyvitamin D_3_ (25OHD) was measured by the IDS-iSYS Multi-Discipline Automated System. PTH was measured by Cobas 411 analyser (Roche Diagnostics International AG, Rotkreuz, Switzerland). Hypolactasia was defined as the presence of the CC genotype of rs4988235 (measured by TaqMan SNP genotyping assay, ThermoFisher Scientific, Waltham, MA, USA). Vitamin K levels: dephosphorylated uncarboxylated matrix-GLA-protein (dp-ucMGP measured by InaKtif MGP Kit with the IDS-iSYS Multi-Discipline Automated System, both Immunodiagnostic Systems Holdings PLC, Sunderland, UK) was used as a surrogate parameter. dp-ucMGP was split into tertiles, and the lowest tertile was used as a reference; tertile 1: lowest value to 411 nmol/L tertile 2: 412 to 516 nmol/L; tertile 3: 517 to highest value). Sarcopenia detected by each index was used as a dependent variable. A stepwise entry approach was selected.

## 3. Results

### 3.1. Characteristics of Study Participants

Patient characteristics and DXA-derived indices per sex are given in Table 2. As expected, most indices showed statistically significant differences between male and female participants.

### 3.2. Sarcopenia Prevalence According to Each DXA-Derived Index and ASM

The use of different DXA-derived parameters and ASM led to a prevalence of sarcopenia between 0.6 and 36.3 percent in all individuals (for details, see Table 3).

Subgroup analysis in both sexes showed a sarcopenia prevalence between 0 to 25.7 percent in females and 0.9 to 50.3 percent in males (details are given in Supplemental Material Table S1). Analysis according to BMI groups revealed the following results: In our cohort, 40.5% of the individuals were normal weight, 38.9% overweight and 20.5% obese, defined according to WHO criteria. Taking a closer look at the prevalence of sarcopenia per each index in three BMI groups revealed that LESMI, AMMI, ASM, ALMI and TSMI mostly recognised normal weight persons as sarcopenic, whereas SMI mostly recognised overweight or obese individuals as sarcopenic. In normal-weight persons, the prevalence of sarcopenia ranged between 15.4 and 100 percent, dependent on the index used. In overweight and obese persons, the prevalence ranged between 0 and 44.6 or 39.0%, respectively (details see Supplemental Material Table S2). Detailed results on sarcopenia prevalence in gender and BMI specific subgroup analysis are given in Supplemental Material Chapter S1.

### 3.3. Sarcopenia Diagnosis Agreement

To get to the basis of the detected discrepancies in sarcopenia prevalence, we first investigated the overlap (Supplemental Material Chapter S2) and agreement of sarcopenia diagnosis between indices. The number of sarcopenic patients identified by increasing numbers of indices are given in Supplemental Material Figure S1.

#### 3.3.1. Comparison of Sarcopenia Diagnosis Agreement of All Investigated Indices

**Figure 2:**
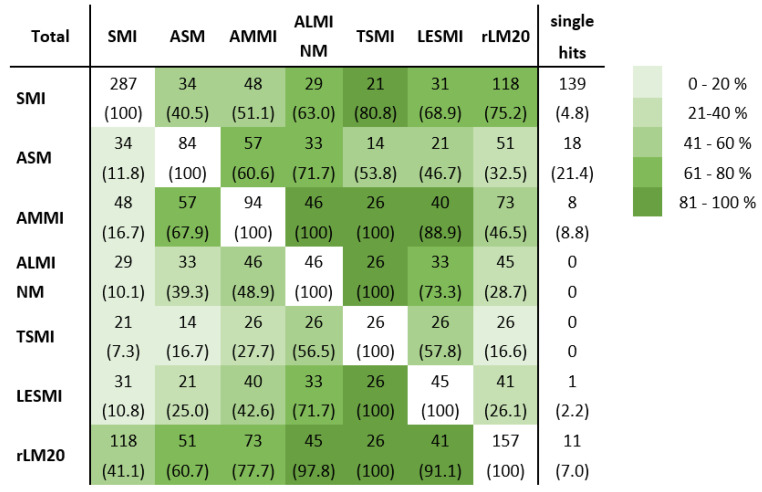
Comparability of sarcopenia diagnosis by use of different DXA-derived indices. SMI, LESMI, rLM20, AMMI, TSMI, ALMI NM and ASM were used in this figure since rLM20 included rLMABC identified sarcopenic persons and ALMI Min in ALMI NM. Each square shows the total numbers of individuals recognised as sarcopenic by the two compared indices (percentage in brackets). The shade of grey indicates the range of overlap as a percentage between the diagnoses of sarcopenia by the two compared indices. The shades range from 0 to 20% accordance (light grey) in steps of 20 percent each up to 81 to 100% accordance (dark grey). DXA: dual-energy X-ray absorptiometry; SMI: skeletal muscle mass index; ASM: appendicular skeletal muscle mass; AMMI: appendicular skeletal muscle mass index; ALMI: appendicular lean mass index; NM: reference values New Mexico; TSMI: total skeletal mass index; LESMI: lower extremity skeletal muscle mass index; rLM20: relative lean mass (20th percentile).

To examine the agreement of sarcopenia diagnosis, we performed pairwise comparisons. We compared the number of sarcopenic individuals identified by each index (see Figure 2) to determine the overlap of individuals diagnosed as sarcopenic. A total of 139 individuals were sarcopenic due to the only weight-adjusted index SMI; 49 individuals were only identified as sarcopenic by one of the height-adjusted parameters or ASM. Substantial agreement was shown between TSMI and LESMI (kappa = 0.721) and with ALMI NM (kappa = 0.71), *p* < 0.001, respectively; ALMI NM with AMMI (kappa = 0.623) and with LESMI (kappa = 0.708), *p* < 0.001, respectively. A moderate level of agreement was found between rLM20 and AMMI (kappa = 0.505, *p* < 0.001), AMMI and LESMI (kappa = 0.535) or ASM (kappa = 0.591), both *p* < 0.001, respectively, as well as ASM and ALMI NM (kappa = 0.468, *p* < 0.001). All other comparisons of sarcopenia diagnosis only showed poor to fair agreement.

#### 3.3.2. Analysis of Factors Affecting Sarcopenia Diagnosis Agreement

To get to the bottom of the variety of levels of sarcopenia diagnosis agreement, we took a closer look at the parameters acting as the origin of the indices, the presence or absence of an adjustment and the type of adjustment.

#### 3.3.3. Underlying Muscle Mass Parameter and Sarcopenia Diagnosis Agreement

##### Skeletal Muscle Mass Based Indices

**Figure 3:**
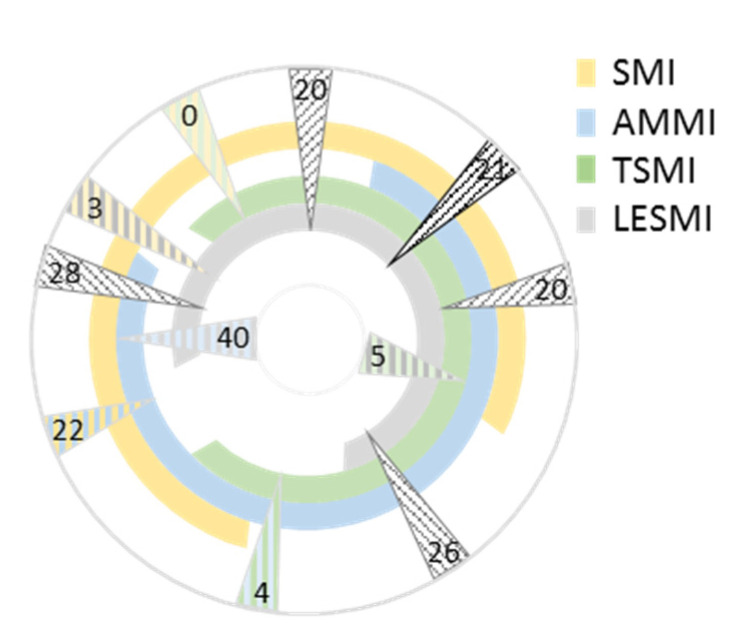
Comparison of the numbers of sarcopenic individuals identified by skeletal muscle mass based indices. Each partial circle depicts an index. The arrows show the compared indices. The darker the arrow, the more indices are compared. The number in the arrows indicate the numbers of individuals identified as sarcopenic by the compared indices. SMI: skeletal muscle mass index; AMMI: appendicular skeletal muscle mass index; TSMI: total skeletal mass index; LESMI: lower extremity skeletal muscle mass index.

Pairwise comparisons revealed an overlap of individuals recognised as sarcopenic in the range of 0 to 40 persons. Comparison of three parameters revealed an overlap of a range from 20 to 28 individuals. In total, 21 individuals were deemed sarcopenic by all four indices (see Figure 3). The strength of agreement of LESMI and TSMI was substantial (kappa = 0.721, *p* < 0.001) and of LESMI and AMMI was moderate (kappa = 0.535, *p* < 0.001). All other pairwise comparisons showed only a slight or fair level of agreement. Only the triple comparison of LESMI, AMMI and TSMI showed a moderate level of agreement (kappa = 0.521, lower bound 0.519 and upper bound 0.522; *p* < 0.001). All four indices showed only a slight level of agreement (kappa = 0.168).

##### Lean Mass-Based Indices

Comparison of the sarcopenic individuals identified by rLM-based indices showed that all individuals with sarcopenia defined by rLMABC are also identified by rLM20.

The comparison of ALMI NM and rLM20 showed that 45 persons were identified by both indices as sarcopenic (fair level of agreement, kappa = 0.388, *p* < 0.001), making it 97.8% of all ALMI NM identified cases or 28.7% of all rLM20-identified cases. No subgroup analysis for gender or BMI for underlying muscle mass parameters was calculated due to the low numbers of persons identified as sarcopenic.

##### Absence or Presence of an Adjustment and Sarcopenia Diagnosis Agreement

**Figure 4:**
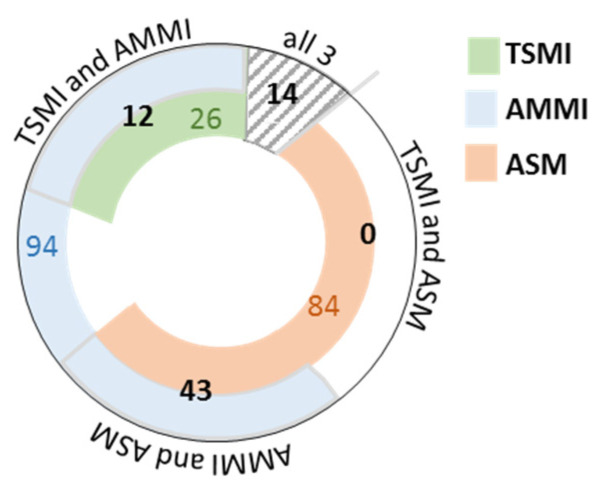
Comparison of the number of sarcopenic individuals identified by ASM-based indices and ASM. Each partial circle depicts an index or ASM. The number in the partial circles indicates the number of individuals recognised as sarcopenic per index. The black numbers reaching over two differently coloured partial circles represent the number of individuals recognised by the two compared parameters. The striped area shows the number of persons identified as sarcopenic, according to all three compared parameters. TSMI: total skeletal mass index; AMMI: appendicular skeletal muscle mass index; ASM: appendicular skeletal muscle mass.

The comparison of the two ASM based indices, AMMI and TSMI, with their underlying unadjusted parameters revealed that only 14 individuals were defined as sarcopenic by all three parameters, 55 individuals by two parameters and 204 individuals only by one parameter used (see Figure 4 for details). Due to its adjustment to weight and the large difference in the prevalence of sarcopenia due to the use of SMI, we did not include SMI in this analysis. The strength of agreement between AMMI and its underlying parameter ASM was moderate (kappa = 0.591, *p* < 0.001), and between TSMI and ASM it was fair (kappa = 0.215, *p* < 0.001). No subgroup analysis for gender and BMI groups for absence or presence of adjustment was calculated due to low numbers of individuals recognised as sarcopenic.

##### Type of Adjustment and Sarcopenia Diagnosis Agreement

A total of 166 persons were defined as sarcopenic by SMI only, 61 by weight adjusted indices only and 120 persons were sarcopenic according to both types of indices. The height-adjusted parameters included AMMI, ALMI NM, rLM20, LESMI and TSMI. ASM, as a non-adjusted parameter, was not included in this analysis.

Comparing only TSMI, LESMI, AMMI and ALMI NM (without rLM20 since it is also adjusted to amount of body fat), the strength of agreement ranges from moderate (LESMI/AMMI: kappa = 0.535, *p* < 0.001) to substantial (TMMI/LESMI: kappa = 0.721; TMMI/ALMI NM: kappa = 0.71; LESMI/ALMI NM: kappa = 0.708; AMMI/ALMINM kappa = 0.623; *p* < 0.001, respectively). Only TSMI and AMMI showed a fair level of agreement, as shown above.

The sex-specific comparison of adjustments revealed a significant difference between the number of persons recognised as sarcopenic with only height, only weight and height and weight-adjusted indices (*p* < 0.001). Height adjusted parameters recognised more women than men as sarcopenic, whereas weight-adjusted parameters deemed more men than women sarcopenic. BMI group-specific comparisons revealed that the individuals identified as sarcopenic by height-adjusted parameters were mainly normal weight, whereas the individuals identified as sarcopenic by SMI were mainly overweight/obese (see Supplemental Material Chapter S3 and Table S3 for details). The number of individuals recognised as sarcopenic in the investigated BMI groups by various adjustments differed significantly (*p* < 0.001).

##### Binary Logistic Regression Models to Determine Nutritional and Clinical Influencing Factors of DXA-Indices

To determine which factors significantly influence ASM, AMMI, ALMI NM, TSMI, LESMI, rLM20 and SMI and whether there are differences, we performed a binary logistic regression analysis. Sex was a significant factor of influence on all indices followed by fat mass, which was only not significant (but associated by trend) when ASM was used as a dependent variable. In addition, BMI was only not significant when SMI was used as a dependent variable, and lifestyle when TSMI was a dependent variable.

**Figure 5:**
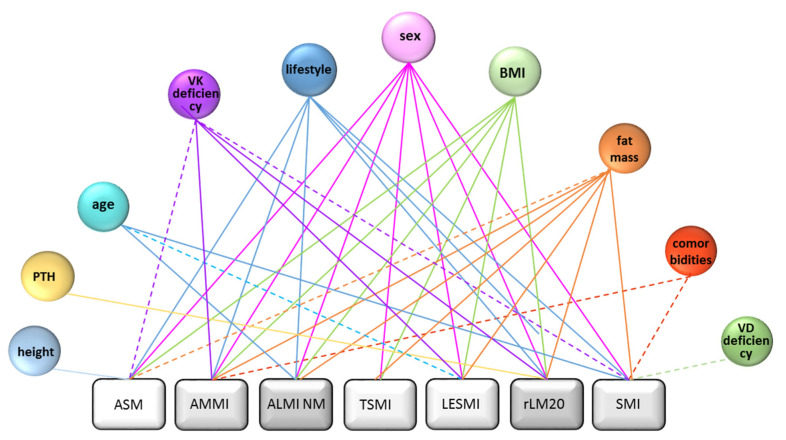
Factors significantly influencing the diagnosis of sarcopenia by each index in our binary logistic regression model. Full lines mark significant associations, dashed lines associations by trend. ASM: appendicular skeletal muscle mass; AMMI: appendicular skeletal muscle mass index; ALMI: appendicular lean mass index; NM: reference values New Mexico; TSMI: total skeletal mass index; LESMI: lower extremity skeletal muscle mass index; rLM20: relative lean mass (20th percentile); SMI: skeletal muscle mass index; PTH: parathyroid hormone; VK: vitamin K; BMI: body mass index. VD: 25-hydroxyvitamin D_3_.

Age showed significant associations with ALMI NM, LESMI and SMI as dependent variables and height only with ASM. The number of comorbidities seems only to be of significant impact for SMI and AMMI. PTH was only significant in our model when influencing rLM20. Vitamin D deficiency was associated with SMI only by trend. Alcohol consumption, smoking and hypolactasia were not significantly associated with any of the investigated indices in our model (see Figure 5). Detailed information regarding *p*-values, relative risk and CI can be found in the supplementary material section. Vitamin K levels, indicated by its surrogate parameter dp-ucMGP, seems to influence ASM, AMMI, ALMI NM, LESMI and rLM20 significantly or at least by trend. For detailed results, see Supplemental Material Table S4.

### 3.4. Evaluation of the Reliability of Sarcopenia Diagnosis

#### 3.4.1. Comparison of Diagnosis Reliability of Investigated Indices

We aimed to examine the reliability of the sarcopenia diagnosis via the investigated parameters by looking closely at the presence of all identifications of sarcopenia in the follow-up data of the consecutive clinical presentation. We gathered follow-up data for the next scheduled visit in 684 persons in total, including data from 308 persons diagnosed with sarcopenia according to at least one DXA-derived index. TSMI and rLM20 were not included in the analysis due to missing follow-up data.

ASM, AMMI, ALMI NM and LESMI showed substantial agreement between both visits; only the agreement of SMIs sarcopenia diagnosis between visits was moderate (see Table 4 for details).

Subgroup analysis according to gender revealed no significant differences in the reliability of sarcopenia diagnosis for ASM, AMMI, ALMI NM and LESMI. Only sarcopenia diagnosis reliability by SMI was significantly lower in females than males (*p* = 0.012) (see Supplemental Material Table S5). Subgroup analysis according to BMI showed significantly lower reliability of diagnosis by AMMI and ALMI NM in individuals with increased BMI compared to normal weight persons. SMI, on the other hand, showed significantly higher reliability of diagnosis in overweight/obese than normal-weight individuals (see Supplemental Material Table S6).

#### 3.4.2. Adjustment and Sarcopenia Diagnosis Reliability

##### Sarcopenia Diagnosis Reliability of Combined Height Adjusted Parameters

Height adjusted parameters included AMMI, ALMI NM and LESMI. In total, 78 persons identified as sarcopenic with follow-up data were included. In 25.6%, the diagnosis of sarcopenia was not confirmed, in 62.8%, the diagnosis was confirmed and in 10.3%, the included parameters showed conflicting results. Since only SMI is weight-adjusted and ASM is the only unadjusted parameter, results for sarcopenia diagnosis reliability as well as sex and BMI specific analysis can be found in Section 3.4.1.

The sex-specific analysis of height-adjusted indices showed no significant differences in agreement between sexes, and the BMI specific analysis of height-adjusted indices showed 78.5% agreement in normal weight persons and 50% agreement in overweight/obese individuals (*p* = 0.039) (for details see Supplemental Material Chapter S4).

##### Sarcopenia Diagnosis Reliability of Adjustment Types

Diagnosis reliability of height-adjusted parameters and unadjusted parameters showed 76.1% agreement, weight-adjusted and unadjusted parameters showed 18.2% agreement and height and weight-adjusted parameters showed 22.8% agreement. The comparison of all three types of adjustment led to a kappa of −0.093 (*p* < 0.001) and is therefore not comparable.

Comparisons of diagnosis reliability in persons recognised by more than one type of index were not performed due to too low numbers. Detailed information regarding sarcopenia diagnosis reliability numbers can be found in the supplemental material. A detailed analysis of sarcopenia diagnosis reliability of each index alone or according to adjustment separately for persons only recognised as sarcopenic by one index (single ID) or by more than one index (multiple ID) can be found in the supplemental material Chapter (S5). Supplemental Material Figure S2 shows the percentage of individuals with multiple IDs together with the percentage of confirmation in the follow up visit. Details of the sex and BMI specific subgroup analysis are given in Supplemental Material Table S7.

## 4. Discussion

The main finding of this study was a large variation in the prevalence of sarcopenia dependent on the index and cut-off values used. A relatively high level of agreement of diagnosis of sarcopenia by only height-adjusted parameters, independent of the use of ALM or ASM as baseline parameters, exists. In comparison, a low level of agreement exists between sarcopenia diagnosis by unadjusted muscle mass parameters and indices adjusted by height only, weight only, and height and body fat. The type of adjustment defines which subjects are recognised as sarcopenic in terms of gender or BMI group. The investigated indices showed comparable reliability and stability of sarcopenia diagnosis in follow-up examinations.

Differences in prevalence of sarcopenia compared to previously published studies can be explained by the method of enrolment, since they are mostly cross-sectional population-based studies compared to our cohort study, including differences in ethnicity and the use of different cut-off points for sarcopenia diagnosis [28]. Errors in the estimation of muscle mass, namely that ASM represents 75% of total skeletal muscle mass and multiplication by 1.33 leads to total skeletal muscle mass, determine the low prevalence of sarcopenia defined by TSMI compared to AMMI [29,30]. The parameter of origin of the investigated indices had no influence on sarcopenia prevalence or diagnosis agreement.

The low level of agreement between differently adjusted indices is in line with the findings of Kemmler et al., but in their study of BIA was used to determine low muscle mass [31]. The low levels of sarcopenia diagnosis agreement between persons identified as sarcopenic in the case of ASM-based indices and ASM can be explained by the lack of adjustment of the latter, not taking into account any parameters determining the amount of muscle mass.

The type of adjustment itself contributes to the low level of agreement between indices. SMI (a weight-adjusted index) recognised mostly overweight persons as sarcopenic and might diagnose sarcopenia earlier than height-adjusted indices in these persons. This is strengthened by the fact that many persons with reduced handgrip strength are defined as sarcopenic only by SMI (kappa = 0.020; *p* < 0.001). In addition, SMI seems to diagnose sarcopenic obesity, a closely related but different disease. The investigated height-adjusted indices recognised sarcopenia mostly in normal-weight persons, clearly underestimating its prevalence in overweight/obese individuals. Due to more skeletal muscle mass but lower muscle quality following the incorporation of fat, in obese people [32], cut-off values for height-adjusted indices might be too low to detect sarcopenia in these individuals.

Our data indicate that neither height nor weight are ideal parameters for the adjustment of DXA- derived muscle mass parameters. No improvement of strength of agreement was gained by the adjustment of ALM with BMI (kappa: 0.003 with LESMI to 0.010 with SMI), since BMI, due to not including body fat percentage, race/ethnicity and the level of activity, does not reflect body composition well [32,33,34]. To address this problem, ALM adjusted to the waist to height ratio, body surface area or in% of weight [35] have been explored but were not satisfying enough to become commonly accepted. rLM is another improvement approach in which linear regression residuals of appendicular muscle mass adjusted for fat mass and height are used [20]. In our study, the level of agreement of rLM20 with SMI was low and only moderate with AMMI. New body composition indices might be better suited for adjustment but have not yet been tested in the diagnosis of sarcopenia. To overcome the flaws of the adjustment now recommended, the use of two adjustments in sarcopenia diagnosis, as investigated for bioelectrical impedance analysis, might be a solution [36].

When using DXA-derived indices to define sarcopenia, the use of cut-off values appropriate for the investigated cohorts in age and ethnicity is crucial. In the context of our cohort, this applies to rLMABC, SMI and LESMI [19,20]. For the last two indices, cut-off values for Caucasians are needed to make them completely comparable. The use of not only sex, but also age-adjusted cut off values, comparable to Z-values in the case of BMD, could be of value.

To determine the reliability of the diagnosis of sarcopenia by each index, we validated our baseline with follow-up results. We assumed that the absence of sarcopenia diagnosis in the follow-up visit indicated that the baseline finding was not reliable since no sarcopenia treatment was taken. Since these are persons at cardiovascular risk, an intervention to decrease it might have been taken, although neither type of dietary intervention nor exercise should impact sarcopenia. Some of the study participants might have changed their lifestyle due to retirement within the two years follow-up interval. The level of agreement between sarcopenia diagnosis in both visits of the indices was comparable. Thus, no index seems to be more reliable than the others.

We noted some gender-specific differences: Dependent on the index used, the prevalence of sarcopenia ranged from 0.9 to 50.3% in men and 0.6 to 36.3% in women. Height adjusted indices recognised more females, whereas for weight-adjusted SMIs, more men were identified as sarcopenic. The reliability of sarcopenia was comparable for all indices, with the exception of SMI, which was significantly more reliable in men than in women.

The lack of vitamins D and K have been associated with sarcopenia prevalence [37,38,39]. Sarcopenia therapy currently combines dietary strategies such as protein supplements and, in the case of sarcopenic obesity, caloric restriction and exercise (most effectively resistance training). This combination is not completely satisfying, and positive effects of the addition of vitamin D or K have been proposed [39,40]. Vitamin K, via enabling of vitamin K dependent proteins to bind calcium by carboxylation, is a known regulator of extra- and intracellular calcium homeostasis. Together with parathyroid hormone, vitamin D and hypolactasia as regulators and modulators of systemic calcium levels, we investigated the dephosphorylated, uncarboxylated Matrix-GLA-protein as a surrogate parameter of vitamin K. Neither vitamin D deficiency nor hypolactasia associated with sarcopenia and parathyroid hormone significantly associated with sarcopenia, as defined by rLM20. Dephosphorylated, uncarboxylated Matrix-GLA-protein presented only at the lowest tertile (representing high vitamin K levels); significant associations with sarcopenia defined by AMMI, LESMI and rLM20, indicated a vitamin K independent pathway.

The study limitations include DXA limitations such as the indirect determination of lean and fat mass, susceptibility to body fluid changes and lack of assessing muscle quality [15,41]. Another limitation is the relatively low mean age of the study participants, which is the reason for suboptimal cut-off values from other studies. Due to our middle-aged cohort, some of the indices also seem to detect presarcopenic stages. In this study, we decided to define sarcopenia only via decreased muscle mass, since our aim was to compare the prevalence of sarcopenia using DXA-derived indices. Due to the low number of persons with reduced hand-grip strength or gait speed, the inclusion of muscle strength information or a simultaneous definition of sarcopenia stages would decrease statistical power and was not within the scope of this work. Another limitation is the use of dephosphorylated, uncarboxylated Matrix-GLA-protein as surrogate parameter instead of a direct measurement of vitamin K metabolites since this does not allow a differentiation between its vitamin K dependent and independent actions.

The strengths of the study are the extensive clinical characterisation of the study participants and the in-depth comparison of nine DXA-derived muscle mass indices with different types of adjustment and cut-off values. The relatively low mean age is not only a limitation but also a strength. In our opinion, the decades of 40 to 60 years provide an age-range useful to detect the early development of sarcopenia, facilitating a higher impact of therapeutic actions.

## 5. Conclusions

Ideally, DXA muscle mass measurements should always be combined with muscle function tests, physical performance information and interpreted in combination with clinical data since the measurement of muscle mass alone will not sufficiently cover a persons’ risk for sarcopenia. An adjustment of DXA-derived muscle mass parameters is necessary to compensate for variations in body composition, but existing adjustments are imperfect. All compared indices, independent of their parameter or origin, were equivalent in their sarcopenia diagnosis reliability in a follow-up, although different persons were recognised as sarcopenic. Further studies are needed to explore new adjustment methods of DXA-derived muscle mass parameters with matching cut-off values to optimise their usefulness in the diagnosis of sarcopenia in both sexes, body composition types and younger individuals with secondary sarcopenia. Consensus definitions on the type and number of indices to be used in clinical studies are warranted to improve the comparability of results.

In study-related approaches, the adjustment of DXA-derived parameters for muscle mass should be selected according to the cohort investigated in terms of BMI and sex distribution, age-range and reference values fitting age and ethnicity. In a clinical setting, a way to bypass this problem might be the combination of two different indices (one weight- and one height-adjusted) for a reliable sarcopenia diagnosis.

## Figures and Tables

**Table 1:** Reference values for low muscle mass in sarcopenia.

Definition of Sarcopenia						
	Unit	Calculation	Cut-Off Values		References	Study/Organisation
			males	females		
SMI	%	ASM/weight[kg] × 100	<29.9	<23.5	Moon 2018 [19]	KNHANESV
LESMI	kg/m^2^	LESM/height^2^	<5.1	<3.7	Moon 2018 [19]	KNHANESV
rLM20	n.a.	ALM adjusted for fat mass	20th percentile		Newman 2003 [20]	Our study
rLMABC	n.a.	and height	−2.29	−1.73	Newman 2003 [20]	ABC Health Study
TSMI	kg/m^2^	TSM/height^2^	9	6	Dufour 2013 [21]	Framingham Heart Study
ALMI	kg/m^2^	ALM/height^2^	<7.26	<5.45	Baumgartner 1998 [22]	New Mexico Elder Health Study
			<6.77	<4.51	Melton 2000 [23]	Rochester Study
AMMI	kg/m^2^	ASM/height^2^	<7	<5.5	Gould 2014 [24]	EWGSOP2
ASM	kg	lean mass arms and legs–bone mass arms and legs	<20	<15	Studenski 2014 [25]	EWGSOP2

SMI: skeletal muscle mass index; ASM [kg]: appendicular skeletal muscle mass, calculation: lean mass arms and legs–bone mass arms and legs [25]; LESMI: lower extremity skeletal muscle mass index; LESM [kg]: lower extremity skeletal muscle mass index, calculation: lean mass legs–bone mass legs [19]; rLM: relative lean mass calculated with linear regression (own cohort), rLM20: 20th percentile used as cut-off values; rLMABC: rLM with cut-off values taken from the ABC Health study; ALM [kg]: appendicular lean mass, calculation: lean mass arms + lean mass legs [15]; TSMI: total skeletal mass index; TSM [kg]: total skeletal mass, calculation: ASM × 1.33 [23]; ALMI: appendicular lean mass index; ALMI NM: ALMI with use of cut-off values of the New Mexico Elder Health Study; ALMI Min: ALMI with use of cut-off values from the Rochester Study; AMMI: appendicular skeletal muscle mass index; kg: kilograms; n. a.: not applicable. KNHANESV: Korean National Health and Nutrition Examination Survey V; EWGSOP2: European Working Group on Sarcopenia in Older People 2nd consensus.

**Table 2:** Description of study participants.

	Male *n* = 340		Female *n* = 448		*p*-Value
Parameter	Mean	SD	Mean	SD	
age [years]	56.9	8.73	56.91	8.03	0.820
BMI [kg/m^2^]	27.5	3.87	25.64	4.74	<0.001
bone mass extremities [kg]	1.69	0.22	1.14	0.17	<0.001
LMI [kg/m^2^]	18.5	1.73	15.25	1.94	<0.001
ALMI-BMI	1.01	0.12	0.73	0.10	<0.001
ALMI [kg/m^2^]	8.71	0.99	6.82	1.04	<0.001
LESM [kg]	18.9	2.75	13.23	2.22	<0.001
TSM [kg]	34.4	4.90	23.16	3.77	<0.001
TSMI [kg/m^2^]	10.9	1.29	8.51	1.33	<0.001
ASM [kg]	25.8	3.68	17.41	2.83	0.002
AMMI [kg/m^2^]	8.18	0.97	6.40	1.00	<0.001
ALM [kg]	27.5	3.83	18.56	2.94	<0.001
SMI [%]	30.0	2.66	25.20	2.62	<0.001
LESMI [kg/m^2^]	5.97	0.71	4.86	0.79	<0.001
rLM	−0.0033	0.9970	−0.0038	0.9979	0.723

BMI: body mass index; LMI: lean mass index; ALMI-BMI: appendicular lean mass index/BMI; ALMI: appendicular lean mass index; LESM: lower extremity skeletal muscle mass; TSM: total skeletal mass; TSMI: total skeletal mass index; ASM: appendicular skeletal muscle mass; AMMI: appendicular skeletal muscle mass index; ALM: appendicular lean mass; SMI: skeletal muscle mass index; LESMI: lower extremity skeletal muscle mass index; rLM: relative lean mass; SD: standard deviation; n: numbers;%: percent; kg: kilograms; m: metres.

**Table 3:** Prevalence of sarcopenia by DXA-derived indices.

	Total		
	Total	Sarcopenia	SP
SMI	788	287	36.3
LESMI	788	45	5.7
AMMI	787	94	11.9
ASM	788	84	10.7
rLM20	788	157	19.9
rLMABC	788	15	1.9
ALMI Min	788	5	0.6
ALMI NM	788	46	5.8
TSMI	788	26	3.3

SP: sarcopenia prevalence in percent. SMI: skeletal muscle mass index; LESMI: lower extremity skeletal muscle mass index; AMMI: appendicular skeletal muscle mass index; ASM: appendicular skeletal muscle mass; rLM20: relative lean mass (20th percentile); rLMABC: relative lean mass (20th percentile Health ABC study); ALMI: appendicular lean mass index; Min: reference values Minnesota; NM: reference values New Mexico; TSMI: total skeletal mass index.

**Table 4:** Percent reliability of sarcopenia diagnosis between baseline and follow-up examination.

	Number	Agreement [%]			
	Total	Yes	No	Kappa *	*p*-Value *
ASM	64	76.6	23.4	0.745	<0.001
AMMI	72	72.2	27.8	0.665	<0.001
ALMI NM	36	61.1	36.1	0.752	<0.001
LESMI	36	58.3	33.3	0.659	<0.001
SMI	241	78.8	21.2	0.585	<0.001

%: percent; * agreement included absence and presence of sarcopenia; ASM: appendicular skeletal muscle mass; AMMI: appendicular skeletal muscle mass index; ALMI: appendicular lean mass index; NM: reference values New Mexico; LESMI: lower extremity skeletal muscle mass index; SMI: skeletal muscle mass index.

## Data Availability

Restrictions apply to the availability of these data. Data are available upon request. To obatin access to the results, a proposal must be submitted to Barbara Obermayer-Pietsch (barbara.obermayer@medunigraz.at). Additional information can be obtained via the Research Management of the Medical University of Graz (tanja.ball@medunigraz.at).

## Supplementary Materials

The following are available online at https://www.mdpi.com/article/10.3390/nu14010186/s1, Figure S1: Numbers of sarcopenic patients identified by increasing numbers of indices. Figure S2: Validation of multiple IDs in the follow-up visit. Table S1: Prevalence of sarcopenia per DXA-based index in both sexes. Table S2: Prevalence of sarcopenia by different DXA-derived indices according to BMI groups. Table S3: Sarcopenic individuals identified by height and/or weight-adjusted parameters per sex or BMI group. Table S4: Detailed results of binary logistic regressions analysis. Table S5: Percent of reliability of sarcopenia diagnosis according to gender. Table S6: Percent of reliability of sarcopenia diagnosis according to BMI subgroups. Table S7: Validation of multiple IDs in the follow-up visit per BMI or sex. Chapter: S1. Detailed results on sarcopenia prevalence in gender and BMI specific subgroup analysis. Chapter S2: Overlap of sarcopenia diagnosis. Chapter S3: Detailed results on the type of adjustment and sarcopenia diagnosis agreement in gender and BMI specific subgroup analysis. Chapter S4: Detailed results of evaluating the reliability of sarcopenia diagnosis—subgroup analysis. Chapter S5: In-depth analysis of sarcopenia diagnosis reliability in “single” and “multiple” IDs.

## Abbreviations

ALMI	appendicular lean mass index
ALMI Min	ALMI with reference values Minnesota
ALMI NM	ALMI with reference values New Mexico
AMMI	appendicular skeletal muscle mass index
ASM	appendicular skeletal muscle mass
LESMI	lower extremity skeletal muscle mass index
rLM20	relative lean mass (20th percentile)
rLMABC	relative lean mass (20th percentile Health ABC study)
SMI	skeletal muscle mass index
