# Supplementary material for: DXA-Derived Indices in the Characterisation of Sarcopenia
Source: Nutrients. 2021 Dec 31;14(1):186. doi: 10.3390/nu14010186 (PMC8747660; doi:10.3390/nu14010186)
Supplement: Supplementary file 1 [file nutrients-14-00186-s001.zip › nutrients-1519231-supplementary.pdf]

Supplemental Material:

S1. Detailed results on sarcopenia prevalence in gender and BMI specific subgroup analysis

Sarcopenia in males ranged from 0.9 to 50.3 percent and in females from 0 to 25.7 percent dependent on DXA-derived muscle mass index used. Sarcopenia prevalence differed significantly in men and women when SMI, LESMI, AMMI, ASM, ALMI NM and Min and TSMI were used for sarcopenia diagnosis (see Table S1 for details).

Table S1: Prevalence of sarcopenia per DXA-based index in both sexes:

	**male**			**female**			p-
	total	Sarcopenia	SP	total	Sarcopenia	SP	value
SMI	340	171	50.3	448	115	25.7	<0.001
LESMI	340	35	10.3	448	10	2.2	<0.001
AMMI	339	30	8.8	448	64	14.3	0.027
ASM	340	13	3.8	448	71	15.8	<0.001
rLM20	340	68	20.0	448	89	19.9	0.963
rLMABC	340	3	0.9	448	12	2.7	0.068
ALMI Min	340	5	1.5	448	0	0.0	0.015
ALMI NM	340	23	6.8	448	23	5.1	0.036
TSMI	340	23	6.8	448	3	0.7	<0.001

BMI: body mass index [kg/m^2]; n: number; SP: sarcopenia prevalence [%]; %: percent. SMI: skeletal muscle mass index; LESMI: lower extremity skeletal muscle mass index; AMMI: appendicular skeletal muscle mass index; ASM: appendicular skeletal muscle mass; rLM20: relative lean mass (20th percentile); rLMABC: relative lean mass (20th percentile Health ABC study); ALMI: appendicular lean mass index; Min: reference values Minnesota; NM: reference values New Mexico; TSMI: total skeletal mass index.

Sarcopenia prevalence in normal weight persons ranged from 16.4 to 100 percent and in overweight individuals from 0 to 44.6 percent dependent on the index used for diagnosis. In obese persons, only SMI, rLM20 and rLMABC were able to detect sarcopenic persons, the prevalence ranged from 6.7 to 39 percent (see Table S2 for details).

Table S2: Prevalence of sarcopenia by different DXA-derived indices according to BMI groups

	Sarcopenia cases per index and BMI groups						
	**Total**	**BMI < 25**		**BMI ≥ 25 - 30**		**BMI ≥ 30**	
	**n**	**n**	**SP**	**n**	**SP**	**n**	**SP**
SMI	287	47	16.4	128	44.6	112	39.0
LESMI	45	36	80.0	9	20.0	0	0.0
AMMI	95	83	87.4	12	12.6	0	0.0
ASM	84	75	89.3	9	10.7	0	0.0
rLM20	157	78	49.7	63	40.1	16	10.2
rLMABC	15	9	60.0	5	33.3	1	6.7
ALMI Min	5	5	100.0	0	0.0	0	0.0
ALMI NM	46	39	84.8	7	15.2	0	0.0
TSMI	26	20	76.9	6	23.1	0	0.0

BMI: body mass index [kg/m^2]; n: number; SP: sarcopenia prevalence [%]; %: percent. SMI: skeletal muscle mass index; LESMI: lower extremity skeletal muscle mass index; AMMI: appendicular skeletal muscle mass index; ASM: appendicular skeletal muscle mass; rLM20: relative lean mass (20th percentile); rLMABC: relative lean mass (20th percentile Health ABC study); ALMI: appendicular lean mass index; Min: reference values Minnesota; NM: reference values New Mexico; TSMI: total skeletal mass index.

S2. Overlap of sarcopenia diagnosis

The differences in sarcopenia prevalence by use of different DXA-derived indices led us to have a look on how many persons were identified as sarcopenic by what number of indices. To determine the overlap of sarcopenia diagnosis we investigated the number of individuals deemed sarcopenic by more than one of the following indices: SMI, LESMI, rLM20, AMMI, TSMI, ALMI NM and ASM in the baseline visit (see figure 3). The rLMABC identified sarcopenic persons are included in rLM20 and ALMI Min in ALMI NM. Only 13 individuals were recognized as sarcopenic by all seven indices, 11 individuals by 6 and 14 individuals by 5 indices. In contrast, 199 individuals were deemed sarcopenic by only one index.

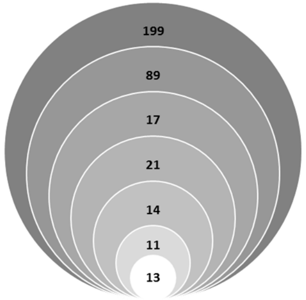

Figure S1: Numbers of sarcopenic patients identified by increasing number of indices: SMI, LESMI, rLM20, AMMI, TSMI, ALMI NM and ASM were used in this figure, since rLMABC identified sarcopenic persons are included in rLM20 and ALMI Min in ALMI NM. The lighter the shade of colour the higher is the number of indices identifying the same patient as sarcopenic. SMI: skeletal muscle mass index; LESMI: lower extremity skeletal muscle mass index; AMMI: appendicular skeletal muscle mass index; ASM: appendicular skeletal muscle mass; rLM20: relative lean mass (20th percentile); ALMI: appendicular lean mass index; NM: reference values New Mexico; TSMI: total skeletal mass index.

S3. Detailed results on type of adjustment and sarcopenia diagnosis agreement in gender and BMI specific subgroup analysis

Sex-specific comparison of adjustments revealed a significant difference between the number of persons recognized as sarcopenic with only height, only weight and height and weight adjusted indices (p<0.001). Height adjusted parameters recognized more women than men as sarcopenic, whereas weight adjusted parameters deemed more men than women sarcopenic (see Table S3 for details).

BMI group specific comparison revealed that the individuals identified as sarcopenic by height adjusted parameters were mainly normal weight whereas the ones identified as sarcopenic by SMI were mainly overweight/obese (see table S3 for details). The number of individuals recognized as sarcopenic in the investigated BMI groups by various adjustments differed significantly (p<0.001).

Table S3: Sarcopenic individuals identified by height and/or weight adjusted parameters per sex or BMI group.

	Sarcopenia defined by parameters adjusted to					
	height		weight		both	
	n=61	%	n=166	%	n=120	%
**Sex**						
**males**	9	14.8	108	65.1	63	52.5
**females**	52	85.2	58	34.9	57	47.5
**BMI [kg/m2]**						

**< 25**	59	96.7	3	1.8	43	35.8
**≥ 25 - 30**	2	3.3	68	41	61	50.8
**≥ 30**	0	0	95	57.2	16	13.3

BMI: body mass index; n: number; %: percent. Height adjusted parameters included AMMI, ALMI NM, rLM20, LESMI and TSMI. AMMI: appendicular skeletal muscle mass index; ALMI: appendicular lean mass index; NM: reference values New Mexico; rLM20: relative lean mass (20th percentile); LESMI: lower extremity skeletal muscle mass index; TSMI: total skeletal mass index.

To determine which factors significantly influence ASM, AMMI, ALMI NM, TSMI, LESMI, rLM20 and SMI and whether there are differences we performed a binary logistic regression analysis. Detailed results of the calculation are given in Table S4.

Table S4: Detailed results of binary logistic regressions analysis

	ASM				AMMI				ALMI NM				TSMI			
	p	RR	95% CI		p	RR	95% CI		p	RR	95% CI		p	RR	95% CI	
			Lower	Upper			Lower	Upper			Lower	Upper			Lower	Upper
sex	<0.0001	103.729	11.814	910.761	0.003	0.071	0.012	0.416	<0.0001	0	0	0.034	<0.0001	4.27E+03	47.629	3.83E+05
age [years]	0.221	0.958	0.895	1.026	0.287	1.032	0.974	1.094	0.040	1.09	1.004	1.185	0.145	0.924	0.830	1.028
lifestyle	0.009	3.689	1.385	9.821	0.001	0.276	0.132	0.577	0.012	0.27	0.096	0.745	0.103	2.517	0.830	7.634
alcohol consumption [drinks/ week]	0.410	0.955	0.856	1.065	0.486	1.030	0.948	1.119	0.470	1.04	0.934	1.158	0.172	0.924	0.825	1.035
smoking [pack years]	0.526	1.015	0.968	1.065	0.285	1.016	0.987	1.046	0.510	1.01	0.974	1.055	0.467	0.982	0.937	1.030
fat mass [kg]	0.057	0.865	0.746	1.004	0.001	1.239	1.086	1.414	0.004	1.38	1.105	1.711	0.045	0.754	0.572	0.994
BMI [kg/m^2]	<0.0001	2.810	1.839	4.294	<0.0001	0.303	0.203	0.452	<0.0001	0.21	0.111	0.398	<0.0001	4.422	1.926	10.154
height [m]	<0.0001	3.36E+13	1.04E+08	1.08E+19	0.432	32.431	0.005	1.92E+05	0.252	0	0	280.1	0.643	45.610	<0.0001	4.84E+08
number of comorbidities	0.720	1.087	0.688	1.718	0.054	1.433	0.994	2.066	0.462	1.19	0.746	1.906	0.127	0.636	0.356	1.138
uc-dpMGP tertiles																
1 (reference)	0.125				0.029				0.186				0.325			
2	0.079	2.732	0.891	8.379	0.009	0.242	0.084	0.700	0.068	0.24	0.051	1.114	0.134	5.578	0.589	52.864
3	0.758	0.828	0.249	2.755	0.451	0.663	0.228	1.931	0.238	0.36	0.064	1.98	0.303	3.212	0.348	29.644

Lactose intolerance	0.558	1.388	0.464	4.149	0.953	1.030	0.389	2.724	0.372	1.94	0.453	8.299	0.231	0.315	0.048	2.084
PTH	0.991	1.000	0.967	1.035	0.662	0.993	0.963	1.025	0.920	1	0.96	1.046	0.238	0.971	0.924	1.020
Vitamin D deficiency	0.726	1.440	0.188	11.036	0.716	0.762	0.176	3.294	0.997	0	0	na	0.997	4.91E+07	<0.0001	na

	LESMI				rLM20				SMI			
	**p**	**RR**	**95% CI**		**p**	**RR**	**95% CI**		**p**	**RR**	**95% CI**	
			**Lower**	**Upper**			**Lower**	**Upper**			**Lower**	**Upper**
sex	<0.0001	890.243	26.058	3.04E+04	<0.0001	36.208	9.305	140.890	<0.0001	7.633	2.854	20.416
age [years]	0.064	0.917	0.837	1.005	0.154	0.969	0.928	1.012	0.013	0.955	0.920	0.990
lifestyle	0.022	3.356	1.195	9.427	<0.0001	2.526	1.544	4.135	0.001	2.016	1.356	2.997
alcohol consumption [drinks/ week]	0.313	0.948	0.854	1.052	0.703	1.012	0.952	1.076	0.163	0.969	0.926	1.013
smoking [pack years]	0.174	0.974	0.937	1.012	0.839	1.002	0.981	1.024	0.676	0.996	0.978	1.014
fat mass [kg]	0.010	0.724	0.568	0.925	<0.0001	0.681	0.611	0.759	<0.0001	0.887	0.830	0.949
BMI [kg/m^2]	<0.0001	4.617	2.275	9.372	<0.0001	2.514	1.957	3.228	0.582	0.963	0.843	1.101
height [m]	0.572	55.903	0.000	6.49E+07	0.224	60.223	0.082	4.43E+04	0.554	0.230	0.002	29.765
number of comorbidities	0.246	0.737	0.440	1.234	0.814	0.970	0.752	1.251	0.088	0.836	0.681	1.027
dp-ucMGP tertiles												
1 (reference)	0.049				0.013				0.728			
2	0.014	11.779	1.632	84.992	0.057	2.189	0.976	4.913	0.546	0.820	0.430	1.563
3	0.103	4.861	0.726	32.538	0.371	0.698	0.317	1.536	0.437	0.760	0.381	1.518
lactose intolerance	0.258	0.405	0.084	1.941	0.289	0.692	0.350	1.367	0.500	0.822	0.464	1.454
PTH	0.403	0.981	0.939	1.026	0.047	1.021	1.000	1.043	0.478	1.007	0.989	1.025
Vitamin D deficiency	0.997	9.47E+07	0.000	na	0.784	0.867	0.314	2.399	0.057	0.443	0.192	1.023

m: meters, kg: kilograms,dp-ucMGP: dephosphorylated, uncarboxylated matrix-GLA-protein; lactose intolerance according to rs4988235 CC genotype, PTH: parathyroid hormone, na: not applicable, p: p-value, RR: relative risk, CI confidence interval, %: percent

S4. Detailed results of evaluation of the reliability of sarcopenia diagnosis – subgroup analysis

Subgroup analysis according to gender revealed no significant differences in reliability of sarcopenia diagnosis for ASM, AMMI, ALMI NM and LESMI. Only sarcopenia diagnosis reliability by SMI is significantly lower in females than males (see Table S5).

Comparison of diagnosis reliability of investigated indices and ASM – subgroup analysis

Table S5: Percent of reliability of sarcopenia diagnosis according to gender

	males			females			
	number	agreement [%]		number	agreement [%]		
	total	yes	no	total	yes	no	p-value
ASM	9	77.8	22.2	55	76.4	23.6	0.926
AMMI	20	60.0	40.0	52	76.9	23.1	0.151
ALMI NM	13	69.2	30.8	18	72.2	27.8	0.856
LESMI	23	69.6	30.4	10	50.0	50.0	0.433
SMI	145	84.1	15.9	95	70.5	29.5	0.012

%: percent; ASM: appendicular skeletal muscle mass; AMMI: appendicular skeletal muscle mass index; ALMI: appendicular lean mass index; NM: reference values New Mexico; LESMI: lower extremity skeletal muscle mass index; SMI: skeletal muscle mass index.

Subgroup analysis according to BMI showed a significantly lower reliability of diagnosis by AMMI and ALMI NM in individuals with increased BMI compared to normal weight persons. SMI on the other hand showed a significantly higher reliability of diagnosis in overweight/obese than normal weight individuals (see Table S6).

Table S6: Percent of reliability of sarcopenia diagnosis according to BMI subgroups

	normal weight			overweight/obese			
		agreement [%]			agreement [%]		
	total	yes	no	total	yes	no	
ASM	55	80.0	20.0	9	55.6	44.4	0.196
AMMI	62	77.4	22.6	10	40.0	60.0	0.023
ALMI NM	27	77.8	22.2	4	25.0	75.0	0.063
LESMI	24	70.8	29.2	9	44.4	55.6	0.230
SMI	32	62.5	37.5	209	81.3	18.7	0.015

%: percent; ASM: appendicular skeletal muscle mass; AMMI: appendicular skeletal muscle mass index; ALMI: appendicular lean mass index; NM: reference values New Mexico; LESMI: lower extremity skeletal muscle mass index; SMI: skeletal muscle mass index.

Adjustment and sarcopenia diagnosis reliability – subgroup analysis

Sex specific analysis of height adjusted indices showed 68.0 % agreement in males and 76.9 % agreement in females (p=0.403). BMI specific analysis of height adjusted indices showed 78.5 % agreement in normal weight persons and 50 % agreement in overweight/obese individuals (p=0.039).

Due to the low number of follow-up data for individuals only recognized as sarcopenic by height adjusted parameters we cannot draw any conclusions, whether an identification of sarcopenia by an index within multiple identifications is more reliable than by a single one. A higher reliability of sarcopenia diagnosis in multiple identifications tends to be present if one uses ASM and SMI.

S5. In depth analysis of sarcopenia diagnosis reliability in "single" and "multiple" IDs

Identification of sarcopenia by only one index ("single IDs")

To examine if the identification of sarcopenia by only one index („single IDs") was a chance finding or not, we had a closer look at 156 persons with follow-up DXA data from the consecutive clinical presentation (visit) according to the study schedule.

In total, we examined follow-up data of 132 persons identified as sarcopenic only by SMI. In 23.5 percent of these persons, the presence of sarcopenia was not detected in the consecutive measurement. 76.5 percent of all single IDs were confirmed in the second presentation (= 0.49, p<0.001). In addition, 81 % of these persons were defined as sarcopenic only by using SMI in the follow-up visit. In the other individuals, at least one additional index identified them as sarcopenic.

In total, 2.3 % normal weight, 44.7 % overweight and 53 % obese persons were identified as sarcopenic using SMI. In 100 % of normal weight (n=2), 84 % of overweight (n=50) and 91.9 % of obese persons, the diagnosis of sarcopenia by SMI only was confirmed by the follow-up examination.

An investigation of sex-specific differences revealed that in 81.8 % of men and only 65.9 % of women the diagnosis of sarcopenia by SMI was confirmed in the follow-up visit (p=0.039).

Identification of sarcopenia by more than one index ("multiple IDs")

To be recognized as sarcopenic, by multiple indices might suggest more reliability of the diagnosis than of the diagnosis per each index. To test this hypothesis, we examined such „multiple IDs" by examining the confirmation of a positive sarcopenia diagnosis in „multiple IDs" per index and per individual. In total, 445 identifications of sarcopenia (in 156 persons) by the examined indices were investigated in a consecutive follow-up visit.

Analysis per index

Looking at each index separately, the finding was confirmed in 79 % of identifications of sarcopenia by SMI (total: n=240) in the follow-up visit (= 0.583, p<0.001). 76 % of identifications of sarcopenia according to ASM (total: n=63) were still sarcopenic in the follow-up visit (= 0.72, p<0.001). 68 % of identifications of sarcopenia by height adjusted parameters (total: n=141) were confirmed in the follow-up examination (68 % of ALMI NM (total: n=34; = 0.703, p<0.001). 72 % of AMMI (total: n=71; = 0.643, p<0.001) and 64 % of LESMI (total: n=36; = 0.690, p<0.001)). In summary, all indices showed the same reproducibility in the follow-up examination.

To explore BMI groups in detail, all height adjusted parameters, without ASM, were combined due to the low numbers of overweight/obese persons identified as sarcopenic by each index. Investigation of the BMI groups separately showed that identifications of sarcopenia by SMI were confirmed in 66.7 % of normal weight persons, in 80 % of overweight and 82.8 % of obese individuals in the follow-up visit. Identifications of sarcopenia by weight adjusted indices showed that 75 % of normal weight and 37.5 % of overweight persons were also identified in the follow-up visit. 79.6 % of normal weight persons and 55.6 % of overweight persons were found sarcopenic by ASM in the baseline as well as the follow-up examination.

The investigation of sex-specific effects on the confirmation of a sarcopenia diagnosis in the follow-up visit revealed that ALMI NM, LESMI and ASM showed no differences in the numbers of confirmations between sexes (ALMI NM: men 64.3 %, women 68.4 %; LESMI: men 62.5 %, women 60 %; ASM: men 75 %, women 78.9 %). Sex-specific differences were seen by investigating all height adjusted indices together: men showed 62.1 % recovery compared to women, who showed 76.7 % recovery in the follow-up visit. Differences between men and women were also noticed in the definition of sarcopenia by AMMI, where the diagnosis of sarcopenia was confirmed in 60 % of men and 84.1 % of women, and by SMI (p=0.053), where in the follow-up visit 91 % of men and 78 % of women were again deemed to be sarcopenic.

Analysis of multiple IDs together

In 70 individuals with „multiple IDs" follow-up data for ALMI NM, AMMI, ASM, SMI and LESMI were available. In 20 % of these persons, 0 % confirmation in the follow-up visit was found. Less than

35 % confirmation was found in 7.1 % of individuals. 50 % confirmation in 20 % of individuals, >60 % in 14.3 and 100 % in 48.6 percent of individuals. Looking at multiple IDs per individual, there was no single index, which was more prone to be not confirmed in the follow-up visit than the others as seen by investigating each index separately (data not shown).

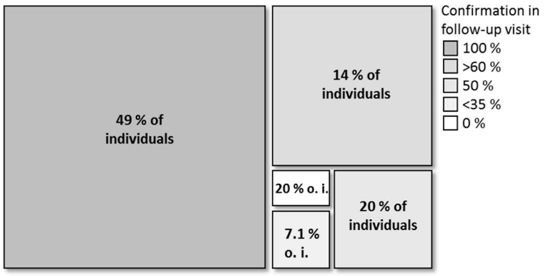

Figure S2: Validation of multiple IDs in the follow-up visit: The boxes indicate the percentage of persons with multiple IDs. The shade of grey indicates in percent, how many of the multiple IDs were confirmed in the follow-up visit. The lighter the shade, the lower the confirmation in percent. Since the individuals had different numbers of multiple IDs, ranging from 2 to 5, 5 confirmation groups were created. %: percent; o. i.: of individuals.

Looking in detail at multiple IDs in BMI subgroups and both genders showed that 100 % confirmation of multiple IDs was achieved in more persons with normal body weight than in overweight persons and in more females than males. More than 60 % recovery was similar in both sexes and BMI subgroups. 50 % confirmation of multiple IDs was achieved in more females than males and in more overweight than normal weight individuals (see Table S7).

Table S7: Validation of multiple IDs in the follow-up visit per BMI or sex:

	**Percentage of persons**		**Percentage of persons**	
**confirmation**	**BMI< 25**	**BMI ≥ 25 - 30**	**males**	**females**
**groups**	n=52	n=16	n=24	n=46
**100 %**	53.8	37.5	41.7	52.2
**> 60 %**	15.4	12.5	12.5	15.2
**50 %**	17.3	31.3	8.3	26.1
**< 35 %**	5.8	12.5	20.8	0.0
**0 %**	7.7	18.8	16.7	6.5

n: number; %: percent

Sarcopenia diagnosis by ASM was found in the follow-up visit at 66.7 % in single IDs and 78.3 % in multiple IDs, and by SMI in 76.5 % in single IDs and 84.4 % in multiple IDs, tending to a higher reliability of sarcopenia diagnosis in multiple IDs.
